# In Vitro and In Vivo Evaluation of Yttria–Niobia-Stabilized Zirconia Sandblasted Surface at Bone–Implant Interface with 3-Dimensional Visualization via Advanced Hard Tissue Clearing

**DOI:** 10.34133/bmr.0377

**Published:** 2026-06-01

**Authors:** Chae-Ryeong Cha, Young-Sung Kim, Satoshi Imazato, Dae-Joon Kim, Yang-Jo Seol, Chang-Joo Park, In-Sung L. Yeo

**Affiliations:** ^1^ Department of Prosthodontics, School of Dentistry and Dental Research Institute, Seoul National University, Seoul, Korea.; ^2^ Department of Periodontology, University of Ulsan College of Medicine and Asan Medical Center, Seoul, Korea.; ^3^ Department of Dental Biomaterials, Graduate School of Dentistry, The University of Osaka, Suita, Osaka, Japan.; ^4^ Faculty of Dentistry, Chulalongkorn University, Bangkok, Thailand.; ^5^ Dental Research Institute, Seoul National University, Seoul, Korea.; ^6^ Department of Periodontology, School of Dentistry and Dental Research Institute, Seoul National University, Seoul, Korea.; ^7^ Division of Oral and Maxillofacial Surgery, Department of Dentistry, College of Medicine, Hanyang University, Seoul, Korea.

## Abstract

Titanium is widely acknowledged as the “gold standard” for dental implants because of its superior biocompatibility and osseointegration capabilities. However, it is limited by the possibility of metal allergies, aesthetic concerns such as gray discoloration visible through the thin gingiva, and the risk of corrosion. To overcome these limitations, metal-free implant materials, particularly yttria–niobia-stabilized tetragonal zirconia polycrystals ((Y,Nb)-TZP), have attracted increasing attention. Zirconia has been noted for its advantages, including promising mechanical properties, biocompatibility, aesthetics, and reduced bacterial adhesion. Despite these findings, comparative studies of sandblasted, large-grit, acid-etched (SLA) titanium and (Y,Nb)-TZP are limited. This study aimed to compare the in vitro osteogenic potential and in vivo osseointegration of sandblasted (Y,Nb)-TZP implants with those of conventional SLA titanium implants using 3-dimensional evaluation. (Y,Nb)-TZP implants demonstrated comparable in vitro and in vivo results to SLA titanium, despite significantly lower surface roughness. Early cell attachment and spreading were observed on (Y,Nb)-TZP, whereas alkaline phosphatase activity and late-stage osteogenic gene expression increased over time in both groups without intergroup differences. In in vivo experiments, no significant between-group differences were observed in bone-to-implant contact, bone area, and dynamic bone remodeling indices. Three-dimensional-based analysis revealed similar overall bone remodeling volumes but distinct spatial distributions of newly formed bone around the implant threads, providing additional insights into the subcortical peri-implant bone area. In conclusion, sandblasted (Y,Nb)-TZP implants exhibited osteogenic potential and osseointegration comparable to those of SLA titanium implants, supporting the potential clinical applicability of (Y,Nb)-TZP as an alternative implant material.

## Introduction

Titanium, owing to its superior biocompatibility and osseointegration capabilities, is widely recognized as the gold standard for dental implant materials [1]. For decades, dental implants have been one of the most effective and predictable treatment approaches to restoring impaired oral function. Various surface modification techniques have been studied, and sandblasted, large-grit, acid-etched (SLA) surface treatment is widely used to optimize surface characteristics and enhance osteoblast proliferation and differentiation [2–4].

However, titanium implants have several limitations. Clinical studies express concerns about the possibility of metal allergies, aesthetic problems such as gray discoloration visible through thin gingiva, and a risk of corrosion [5,6]. To overcome these challenges, metal-free implant materials have attracted increasing attention, and yttria (Y_2_O_3_)-stabilized zirconia has been introduced. Its advantages include promising mechanical properties, biocompatibility, aesthetics, and reduced bacterial adhesion [5,7–9]. Nevertheless, its susceptibility to fracture and the potential for clinical failure, especially from low-temperature degradation in the oral environment, remained substantial concerns [10,11].

The yttria–niobia-stabilized tetragonal zirconia polycrystal ((Y,Nb)-TZP), which incorporate both yttrium (Y) and niobium (Nb), has emerged as a promising solution for these inherent limitations. This novel composition enhanced resistance to low-temperature degradation and fracture, demonstrating robust mechanical stability [12]. Furthermore, as SLA surface treatment might not be optimal for zirconia owing to its high resistance to acid etching and the subsequent reduction in fracture load, various surface modification techniques have been investigated to enhance the biological responses essential for osseointegration [13–16]. These advancements effectively address the drawbacks of the earlier zirconia implants, indicating a clinical performance that may be comparable to or surpass that of conventional titanium implants [17].

Despite these findings, comparative studies of SLA titanium and (Y,Nb)-TZP remain limited [18]. This lack of a comprehensive understanding impedes the adoption of this prospective material and necessitates a thorough comparison of the 2 materials across diverse parameters, including cellular responses and osseointegration. Furthermore, although traditional histological sections provide valuable assessments of the bone–implant interface, their inherent 2-dimensional (2D) nature may preclude precise quantitative analysis by obscuring critical 3-dimensional (3D) information. Bone-clearing techniques, particularly the polyethylene glycol (PEG)-associated solvent system (PEGASOS), offer an innovative approach for the volumetric analysis of dynamic bone formation, yielding deeper biological insights [19,20].

This study aimed to evaluate the in vitro and in vivo characteristics of SLA titanium and (Y,Nb)-TZP implants. Specifically, advanced 3D-based analyses of the bone–implant interface were conducted using the PEGASOS bone-clearing method. Through rigorous evaluation of the in vitro and in vivo characteristics of (Y,Nb)-TZP against the established SLA titanium, this study is expected to provide robust and balanced scientific evidence to guide future implant material selection and development.

## Materials and Methods

### Experimental design

**Fig. 1.**
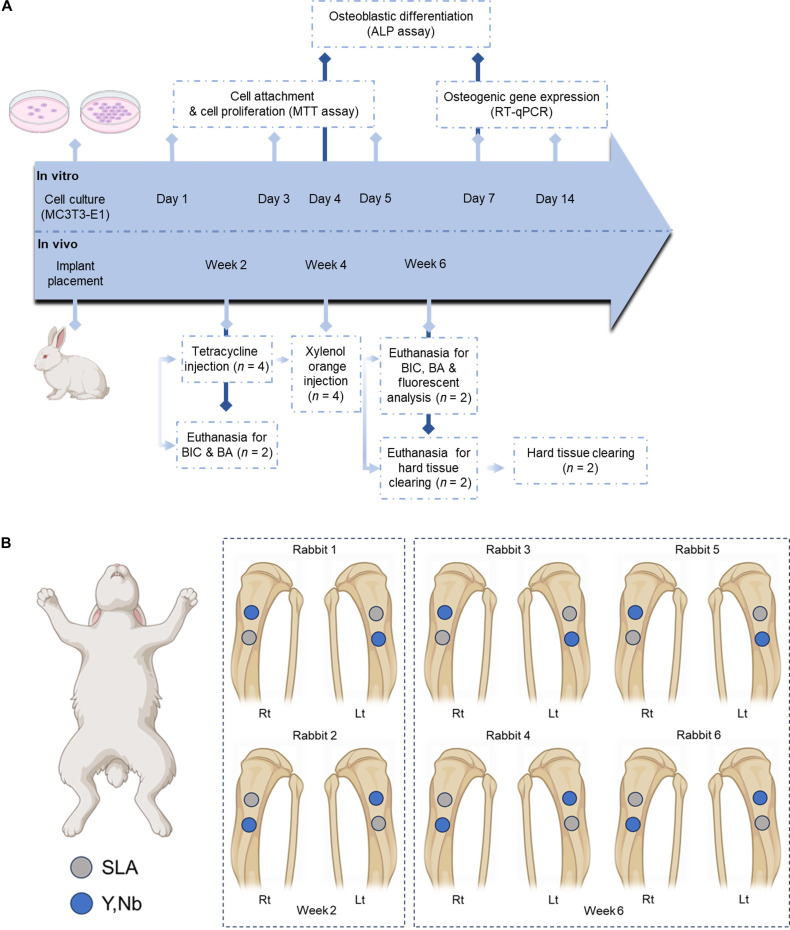
Experimental design and implant distribution in the rabbit tibia model. (A) Overview of in vitro and in vivo experiment procedures. (B) Schematic illustration of implant placement in 6 New Zealand white rabbits. Each rabbit received implants in both the right (Rt) and left (Lt) tibiae, with one sandblasted, large-grit, acid-etched (SLA) titanium implant (gray circles, control) and one yttria–niobia-stabilized tetragonal zirconia polycrystal ((Y,Nb)-TZP) implant (blue circles, experimental) placed per tibia following a predetermined alternating sequence to minimize positional bias. Rabbits 1 and 2 were sacrificed at 2 weeks postimplantation and were evaluated only for bone-to-implant contact (BIC) and bone area (BA) without fluorescent labeling. Rabbits 3 and 4 were sacrificed at 6 weeks and underwent fluorescent labeling prior to sacrifice to allow both histomorphometric (BIC and BA) and dynamic bone formation analyses. Rabbits 5 and 6 were sacrificed at 6 weeks after fluorescent labeling and were used for 3-dimensional (3D) fluorescence-based bone analysis.

This study hypothesized that (Y,Nb)-TZP implants would present biocompatibility and peri-implant bone responses comparable to those of SLA titanium implants and that 3D hard tissue clearing would allow the visualization and comparison of subcortical peri-implant bone formation. To test this hypothesis, results of in vitro experiments including the surface characteristics of both materials, cell attachment, proliferation, osteoblastic differentiation, and osteogenic gene expression of preosteoblastic cells were analyzed. For in vivo evaluation, implants were placed in the tibiae of rabbits, and peri-implant bone responses were assessed using conventional histomorphometric parameters. In addition, sequential fluorochrome labeling combined with a 3D hard-tissue-clearing technique was employed to visualize and quantitatively evaluate dynamic peri-implant bone formation in 3 dimensions (Fig. 1A).

### Specimen preparation

Both implant- and disc-shaped specimens were prepared to evaluate SLA titanium and (Y,Nb)-TZP comparatively. Thirty implants were fabricated for in vivo and in vitro experiments in total, comprising 15 commercially available SLA titanium implants (Deep Implant System, Seongnam, Korea; Ø3.4 mm × 8 mm) and 15 (Y,Nb)-TZP implants (Vatech MCIS, Suwon, Korea), respectively. The composition of zirconia powder was 90 to 92 mol% ZrO_2_, 4 to 5 mol% Y_2_O_3_, 4 to 5 mol% Nb_2_O_5_, and <1 mol% other oxides. With this powder, the zirconia implants were produced by injection molding, followed by sintering at 1,400 °C for 2 h, and subsequently sandblasted with 125-μm Al_2_O_3_ at 4 bar. In addition, zirconia discs (10-mm diameter and 1-mm thickness) of the same composition were produced by uniaxial pressing at 60 MPa and cold isostatic pressing at 186 MPa, after which identical sintering and sandblasting procedures were conducted. SLA titanium discs were also fabricated as control specimens, and all of the prepared discs were used for surface characterization and in vitro cellular assays.

### Surface roughness and characteristics

To determine the surface characteristics, 3 specimens from each SLA titanium and (Y,Nb)-TZP group were analyzed at 3 randomly selected points per specimen. To perform a qualitative evaluation, field-emission scanning electron microscopy (SEM; Hitachi S-4700, Hitachi, Tokyo, Japan) was used to observe the overall surface morphology, while the chemical composition of each surface was examined by energy-dispersive spectroscopy (EDS; Sigma Probe, Thermo VG, East Grinstead, UK). In addition, the surface roughness (*S*_a_) was measured for quantitative analysis using a confocal laser scanning microscope (CLSM; LSM 800, Carl Zeiss AG, Oberkochen, Germany).

### Cell culture

The MC3T3-E1 murine preosteoblastic cell line (American Type Culture Collection, Manassas, VA, USA) was cultured in α-minimum essential medium (Thermo Fisher Scientific, Waltham, MA, USA) containing 10% fetal bovine serum (Sigma-Aldrich, St. Louis, MO, USA) and 1% penicillin–streptomycin. To induce osteogenic differentiation, the medium was further supplemented with 10 mM β-glycerophosphate and 50 μg/ml ascorbic acid. Cells were cultured at 37 °C in a humidified incubator with 5% CO_2_, and the culture medium was renewed every third day. For experimental procedures, MC3T3-E1 cells were plated onto disc specimens at a density of 1 × 10^4^ cells per well in 24-well culture plates (SPL Life Sciences, Pocheon, Korea).

### Cell attachment

Preosteoblastic cells on disc specimens (*n* = 3) were analyzed at 1, 3, and 5 d after seeding to evaluate early cell attachment. After removing the culture medium, cells were fixed in 4% formaldehyde (Thermo Fisher Scientific) for 30 min and rinsed twice with Dulbecco’s phosphate-buffered saline (DPBS; Thermo Fisher Scientific). The cells were then permeabilized with 0.1% Triton X-100 (Thermo Fisher Scientific) for 15 min and followed by an additional DPBS wash. Subsequently, they were blocked with 1% bovine serum albumin (Thermo Fisher Scientific) for 30 min and stained with fluorescent probes: Alexa Fluor 488 phalloidin (Invitrogen, Carlsbad, CA, USA) for F-actin and 4′,6-diamidino-2-phenylindole (Invitrogen) for nuclear visualization. The stained discs were mounted on confocal dishes using ProLong Gold Antifade Mountant (Thermo Fisher Scientific) and allowed to settle overnight. Fluorescence imaging was performed using a CLSM (LSM 980, Carl Zeiss AG, Oberkochen, Germany) to observe the cytoskeletal organization and cellular spreading. For quantitative evaluation, each disc surface was divided into 4 regions, and one representative field per region was captured at ×20 magnification. The ImageJ software (National Institutes of Health, Bethesda, MD, USA) was used to analyze the cell attachment characteristics.

### Cell proliferation

Cell proliferation and metabolic activity were evaluated using the MTT assay (TOX-1, Sigma-Aldrich) on 3 disc specimens per group (*n* = 3) at 1, 3, and 5 d after cell seeding. This colorimetric method quantifies the mitochondrial reduction of the yellow tetrazolium salt, 3-(4,5-dimethylthiazol-2-yl)-2,5-diphenyltetrazolium bromide. This reduction yielded purple formazan crystals in metabolically active cells. A 5 mg/ml MTT solution was added to the culture medium and incubated for 2 h at 37 °C under 5% CO_2_. The supernatant was then carefully removed, and the resulting formazan was dissolved using 0.5 ml of 10% dimethyl sulfoxide in isopropanol. The dissolved solutions were transferred to a 96-well plate (SPL Life Sciences), and duplicate optical density readings were obtained for each disc. The proliferation rate was assessed by measuring the optical density at 570 nm using a microplate reader (Epoch2, BioTek, Winooski, VT, USA).

### Osteoblastic differentiation

Osteogenic differentiation was evaluated by quantifying intracellular alkaline phosphatase (ALP) activity 4 and 7 d after cell seeding. Cells were cultured on 3 disc specimens per group (*n* = 3) in 24-well plates. After incubation, the cell layers were washed with phosphate-buffered saline (PBS) and lysed with Triton X-100 to extract intracellular enzymes. The resulting cell lysates were transferred into 96-well plates for analysis. ALP activity was measured using a fluorometric assay kit (MAK530, Sigma-Aldrich) that detects the enzymatic conversion of 4-methylumbelliferyl phosphate to the fluorescent product 4-methylumbelliferone. According to the manufacturer’s instructions, 90 μl of the working reagent was added to each well and incubated for 20 min at room temperature. Fluorescence intensity (excitation, 360 nm; emission, 450 nm) was recorded using a multimode microplate reader (Synergy H1, BioTek, Winooski, VT, USA). ALP activity was calculated from a standard curve generated using known concentrations of 4-methylumbelliferone.

### Reverse transcription quantitative real-time polymerase chain reaction

To quantify the gene expression patterns related to osteogenic differentiation, total RNA was extracted from MC3T3-E1 cells cultured for 7 and 14 d using TRIzol reagent (Invitrogen). The isolated RNA (1 μg) then underwent reverse transcription into complementary DNA using the PrimeScript RT Master Mix (TaKaRa Bio Inc., Shiga, Japan). Quantitative polymerase chain reaction was subsequently performed utilizing TB Green Premix Ex Taq II (Tli RNaseH Plus, TaKaRa Bio Inc.) for detection. All amplification reactions were performed using QuantStudio 3 Real-time PCR System (Applied Biosystems, Foster City, CA, USA). The transcript levels of osteogenic markers, including ALP, collagen type I alpha 1 (COL1A1), osteocalcin (OCN), and runt-related transcription factor 2 (RUNX2), were evaluated. Relative gene expression was normalized to that of the endogenous control gene glyceraldehyde-3-phosphate dehydrogenase. The comparative cycle threshold (2^−ΔΔCt^) method was employed to determine the fold changes in gene expression.

### In vivo surgery

All animal procedures were approved by the Institutional Animal Care and Use Committee of Cronex (CRONEX-IACUC: 202503009) and conducted in accordance with the ARRIVE 2.0 guidelines. Six male New Zealand white rabbits (approximately 2.5 kg each) were used in this study. The experimental unit was an individual implant, with the implants placed in each rabbit.

Prior to surgery, general anesthesia was achieved through an intramuscular injection of xylazine hydrochloride (4.664 mg/kg; Rompun, Bayer, Leverkusen, Germany) combined with intravenous administration of tiletamine hydrochloride (5 mg/kg) and zolazepam hydrochloride (5 mg/kg; Zoletil 50, Virbac, Carros, France). Surgical fields on both hind limbs were shaved and disinfected with povidone–iodine. A local anesthetic (2% lidocaine with epinephrine, 1:100,000; Huons, Seongnam, Korea) was injected into the incision site.

A straight incision approximately 3 to 5 cm in length was made in the medial aspect of the tibia. After reflecting the skin and fascia using a periosteal elevator, the cortical surface was exposed. Implant osteotomies were sequentially prepared using drills of 2.0-, 2.5-, and 3.2-mm diameters, with the drilling depth limited to the upper cortical plate. Each tibia received 2 different types of implants (SLA titanium and (Y,Nb)-TZP) arranged in a split-plot layout to reduce animal use. Importantly, this within-subject experimental design inherently accounted for interanimal biological variation by exposing both implant types to an identical systemic environment. The rabbit tibia was selected as the in vivo model for this study because it is a widely recognized, standardized model for assessing the biological responses of dental biomaterials, and its high bone turnover rate allows for the efficient evaluation of early bone healing and osseointegration [21]. Implant allocation was performed alternately within each tibia according to a predetermined sequence to minimize positional bias. The implants were positioned such that the boundary between the macro- and micro-threaded regions was aligned with the cortical surface. Soft tissues were closed in layers using 4-0 Vicryl (Coated Vicryl, Ethicon, Raritan, NJ, USA) and 4-0 nylon sutures (Ailee, Busan, Korea). Postoperative analgesia was provided in accordance with the institutional guidelines, and the animals were monitored for signs of pain or distress. After the surgery, each rabbit was housed separately during recovery to minimize stress and movement. No predefined inclusion or exclusion criteria were applied, and no animals or implants were excluded from the analysis. The sample size was determined based on the specific sample allocation from a previous comparable study, which utilized 2 rabbits per time point to compare 2 different groups, yielding 4 implants per group [22]. This approach was adopted alongside strict ethical considerations to minimize animal use in accordance with the 3Rs principles (Fig. 1B).

### Fluorescent labeling and histomorphometric analysis

Two weeks postimplantation, 2 rabbits were humanely euthanized by intravenous administration of potassium chloride (15 mg/kg) under deep general anesthesia. Tibiae were collected for preliminary histomorphometric assessments without fluorescent bone labeling.

Sequential fluorescent bone labeling was performed on the other 4 rabbits designated for euthanasia at 6 weeks postoperatively. Tetracycline (63 mg, 10 mg/ml in 0.45% PBS; Cytiva, Marlborough, MA, USA) was injected intramuscularly 2 weeks postoperatively. Subsequently, xylenol orange (230 mg, 100 mg/ml in 1% NaHCO_3_ buffer; Sigma-Aldrich) was administered intramuscularly at 4 weeks. These 4 fluorescently labeled rabbits were euthanized using the same intravenous potassium chloride injection method 6 weeks postimplantation.

All tibiae harvested from the 2-week group and 2 rabbits from the 6-week group underwent immediate fixation in 10% neutral-buffered formalin and were subsequently embedded in resin without decalcification. Sections of 50-μm thickness were prepared from these samples. For the 2-week samples, the prepared sections were stained with Goldner’s Masson trichrome to delineate the bone-to-implant contact (BIC) and quantify the bone area (BA). Histological specimens were examined under a light microscope (DM2700M, Leica Microsystems, Wetzlar, Germany) at ×50 magnification. Images from 3 consecutive implant threads were captured and analyzed using the ImageJ software (National Institutes of Health).

For the 2 fluorescently labeled 6-week specimens that had undergone sectioning, dynamic histomorphometric parameters were first evaluated. Fluorescent labels that specifically bind to newly formed bone were used to assess bone formation dynamics by marking active mineralization fronts at distinct time points. Fluorescence microscopy (DMi8, Leica Microsystems) of these sections was performed to quantify the mineral apposition rate (MAR), mineralizing surface per bone surface (MS/BS), and bone formation rate per bone surface (BFR/BS) [23]. Following fluorescence imaging, the same sections were stained with Goldner’s Masson trichrome to further evaluate bone histology, including BIC and BA. Light microscopy and ImageJ analyses were conducted using the same parameters.

MAR was calculated by dividing the interlabeling distance (Int.Lbl.dst) between the tetracycline and xylenol orange fluorescent lines by the time interval of the labeling period (Int.Lbl.time), which was 2 weeks (14 d) between the 2 biomarker administrations in this study (MAR = Int.Lbl.dst/Int.Lbl.time) [24].

To quantify MS/BS, the total length of the biomarker detected at 2 weeks (single-labeled surface [sLS]) and 4 weeks (double-labeled surface [dLS]) was measured using the ImageJ software (National Institutes of Health). MS/BS was then determined as the sum of sLS and half of dLS and expressed as a percentage of the total bone surface area (BS) (MS/BS (%) = (sLS + 0.5 × dLS)/BS × 100) [25]. BFR/BS was subsequently calculated as the product of MAR and MS/BS (BFR/BS = MAR × (MS/BS)) [23].

### Micro-computed tomography imaging for 3D reconstruction

For the remaining 2 fluorescently labeled rabbits from the 6-week group, the tibiae were processed for advanced 3D analysis. Prior to the in vivo analysis, the rabbits underwent transcardial perfusion, involving an initial perfusion of 1,050 ml of 1× PBS, followed by 1,050 ml of 4% paraformaldehyde containing 10% sucrose in PBS.

Harvested tibiae were subsequently subjected to micro-computed tomography (micro-CT) scanning. Each tibia, containing both SLA titanium and (Y,Nb)-TZP implants, was carefully placed in a 50-ml tube for image acquisition. CT was performed using a Skyscan 1273 device (Bruker, Billerica, MA, USA). Specifically, SLA-titanium-implanted regions were scanned at a 120-kV voltage and a 125-μA current, while (Y,Nb)-TZP-implanted regions were scanned at a 130-kV voltage and a 115-μA current. Both implant types were scanned with a 15-μm voxel size. Three-dimensional image reconstruction was conducted using the Imaris 9.0 software (Bitplane, AG, Zurich, Switzerland).

### PEGASOS hard-tissue-clearing process

Following micro-CT scanning, the samples underwent a modified immersion-based hard-tissue-clearing protocol specifically adapted for hard tissues [20]. Decalcification was omitted in this protocol to prevent the potential loss of fluorescent signals from the bone labels. After fixing with 4% paraformaldehyde fixation at room temperature for 12 h, the samples were decolorized. Owing to the larger size of the tibial samples compared to those in the original protocol, all subsequent reagent volumes were doubled to ensure effective penetration and processing, and all solutions were changed every day.

Decolorization was performed in a 2-step process at 37 °C under constant shaking. First, samples were decolorized with 25% (v/v in H_2_O) Quadrol (*N*,*N*,*N*′,*N*′-tetrakis(2-hydroxypropyl)ethylenediamine; Sigma-Aldrich, Cat. No. 122262) for 4 d, followed by treatment with a 5% (v/v in H_2_O) ammonia solution (Supelco/Sigma-Aldrich, Cat. No. 105432).

Serial delipidation and subsequent dehydration were performed. Samples were placed in gradient delipidation solutions for 4 d, maintaining constant shaking at 37 °C. These solutions comprised 30% tB (70% v/v H_2_O, 27% v/v *tert*-butanol [tB; Sigma-Aldrich, Cat. No. 360538]), 50% tB (50% v/v H_2_O, 47% v/v tB), and 70% tB (30% v/v H_2_O, 67% v/v tB). Each delipidation solution was supplemented with 3% (w/v) quadrupole (Sigma-Aldrich) to adjust the pH above 9.5. Following delipidation, the samples were dehydrated for 4 d in a tB–PEG solution composed of 70% v/v tB and 27% v/v poly(ethylene glycol) methyl ether methacrylate average Mn500 (PEGMMA500; Sigma-Aldrich, Cat. No. 409529) and 3% w/v Quadrol (Sigma-Aldrich), also at 37 °C.

Finally, for the clearing process, the samples were immersed in a BB–PEG clearing medium (refractive index, 1.543). The culture medium was prepared by mixing 75% v/v benzyl benzoate (BB; Sigma-Aldrich, Cat. No. B6630) and 25% v/v PEGMMA500 (Sigma-Aldrich) and supplemented with 3% (w/v) Quadrol (Sigma-Aldrich). Samples were maintained in this medium at 37 °C for at least 2 d until complete transparency was achieved. The cleaned samples were then preserved in the same BB–PEG clearing medium at room temperature for storage and subsequent detailed 3D visualization and fluorescent observation of the bone–implant interface.

### Image acquisition and 3D reconstruction

Following the hard-tissue-clearing procedure, 3D imaging of the cleared tibiae was performed using a light-sheet fluorescence microscope (Lightsheet 7, Carl Zeiss Microscopy GmbH, Jena, Germany). Light-sheet fluorescence microscopy (LSFM) was chosen because of its advantages in rapidly imaging large, optically cleared biological samples while minimizing phototoxicity and photobleaching compared to conventional fluorescence microscopy. This approach enables comprehensive 3D visualization of bone structures and fluorescent labels without compromising sample integrity.

The cleaned samples were mounted on an appropriate sample holder and immersed in BB–PEG clearing medium. Imaging was conducted using identical ×5/0.16 detection and ×5/0.1 illumination objectives. The laser excitation was set at 488 nm for tetracycline (emission filter BP 505-545, EF1) and 561 nm for xylenol orange (emission filter BP 575-615, EF2). The camera beam splitter was configured as an SBS LP 560. Optical z-stacks were acquired with a step size of 10 μm and a 10% tile overlap, using an exposure time of 100 ms per optical section. Images were captured at a pixel size of 0.92 × 0.92 μm with a pco.edge 4.2 camera controlled by the ZEN acquisition software.

The raw image datasets were subsequently processed for 3D reconstruction and visualization. The final 3D reconstruction and quantitative analyses were performed using Imaris 9.0, as described in the previous section, (Bitplane).

### Three-dimensional image-based quantitative analysis of bone volume

Quantitative analysis was performed using the Imaris 9.0 software (Bitplane) on the 3D reconstructed images obtained from LSFM of the cleared samples. A region of interest (ROI) was defined as a 500 × 500 × 500 μm stack within the thread grooves of each implant, and the size was selected to encompass a representative portion of the interthread region. Within these ROIs, key quantitative parameters were evaluated through surface-based quantification using the “Statistics” function. Specifically, the bone volume-to-total volume ratio was quantified by segmenting the bone tissue directly from the LSFM data and calculated as the volume of the selected bone region divided by the total volume of the ROI. Further, the volumes of the fluorescent signals from both tetracycline and xylenol orange were quantified within the defined ROIs. For each sample, at least 3 ROIs exhibiting active peri-implant bone formation were selected within the threaded grooves.

### Statistical analysis

All in vitro experiments, including cell attachment, proliferation, and differentiation assays (ALP and reverse transcription quantitative real-time polymerase chain reaction [RT-qPCR]), were performed in independent triplicates (*n* = 3) to ensure reproducibility and statistical reliability, following widely established in vitro evaluation protocols for dental implant materials [17]. All quantitative data are presented as mean ± standard deviation. Statistical analyses were performed using the R software (version 4.5.2, R Foundation for Statistical Computing, Vienna, Austria). The normality of the data distribution was verified using the Shapiro–Wilk test. Differences among groups were assessed using one-way analysis of variance (ANOVA). When statistical significance was detected by ANOVA, post hoc comparison was conducted using Tukey’s honestly significant difference test to identify specific intergroup differences. The level of statistical significance was set at *P* < 0.05. Different levels of statistical significance are indicated as follows: *P < 0.05, **P < 0.01, and ***P < 0.001.

## Results

### Surface roughness and characteristics

**Fig. 2.**
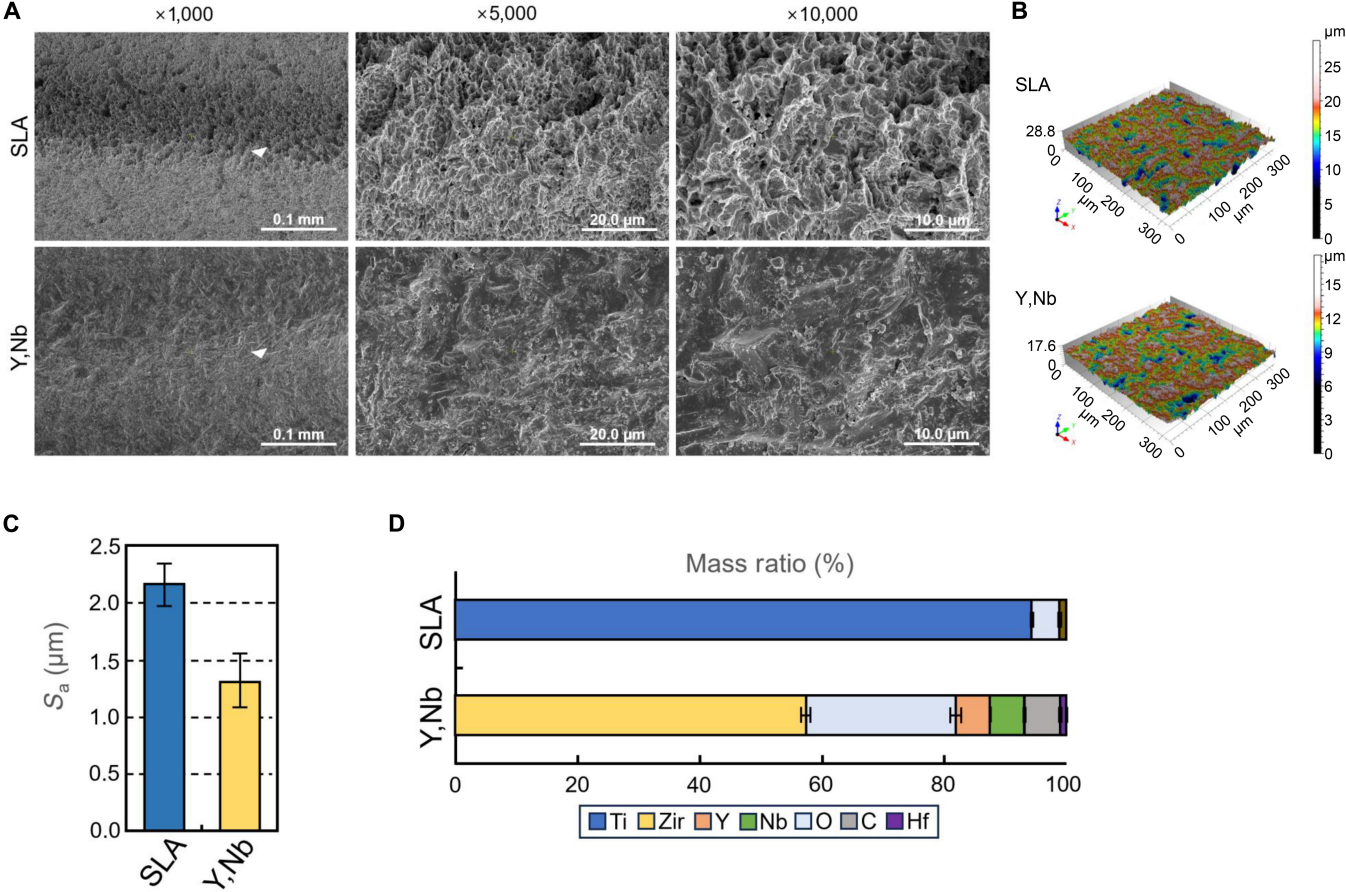
Surface topography and elemental composition of sandblasted, large-grit, acid-etched (SLA) titanium and yttria–niobia-stabilized tetragonal zirconia polycrystal ((Y,Nb)-TZP) implants. (A) Field-emission scanning electron microscopy (SEM) images show the surface morphology of SLA titanium (top) and (Y,Nb)-TZP (bottom) at different magnifications, with the top of the implant thread indicated by white triangles. (B) Three-dimensional surface topography maps obtained by a confocal laser scanning microscope (CLSM) are presented. (C) Quantitative surface roughness (*S*_a_) values are expressed as mean ± standard deviation (SD) (*n* = 3). (D) The elemental compositions of the implant surfaces were analyzed by energy-dispersive x-ray spectroscopy and are presented as mean ± SD (*n* = 3).

SEM, CLSM, and EDS analyses were performed to characterize the surface topography and elemental composition of SLA titanium and (Y,Nb)-TZP. SEM revealed the highly distinguishable surface morphologies of the 2 materials. The SLA titanium surface exhibited a typical microporous, honeycomb-like pattern generated by sandblasting and acid etching, whereas the sandblasted (Y,Nb)-TZP exhibited a more irregular and granular surface configuration (Fig. 2A). These qualitative observations were consistent with quantitative surface analysis using the CLSM, which demonstrated significantly lower average surface roughness values (*S*_a_) for (Y,Nb)-TZP (1.0 to 1.6 μm) compared with those of SLA titanium (2.0 to 2.4 μm), indicating that (Y,Nb)-TZP had a comparatively smoother topography (Fig. 2B and C). Furthermore, EDS verified the elemental composition of each specimen, with titanium being the dominant element in the SLA titanium group, whereas most zirconium and distinct peaks corresponding to yttrium and niobium were identified in the (Y,Nb)-TZP samples (Fig. 2D).

### Cell attachment

**Fig. 3.**
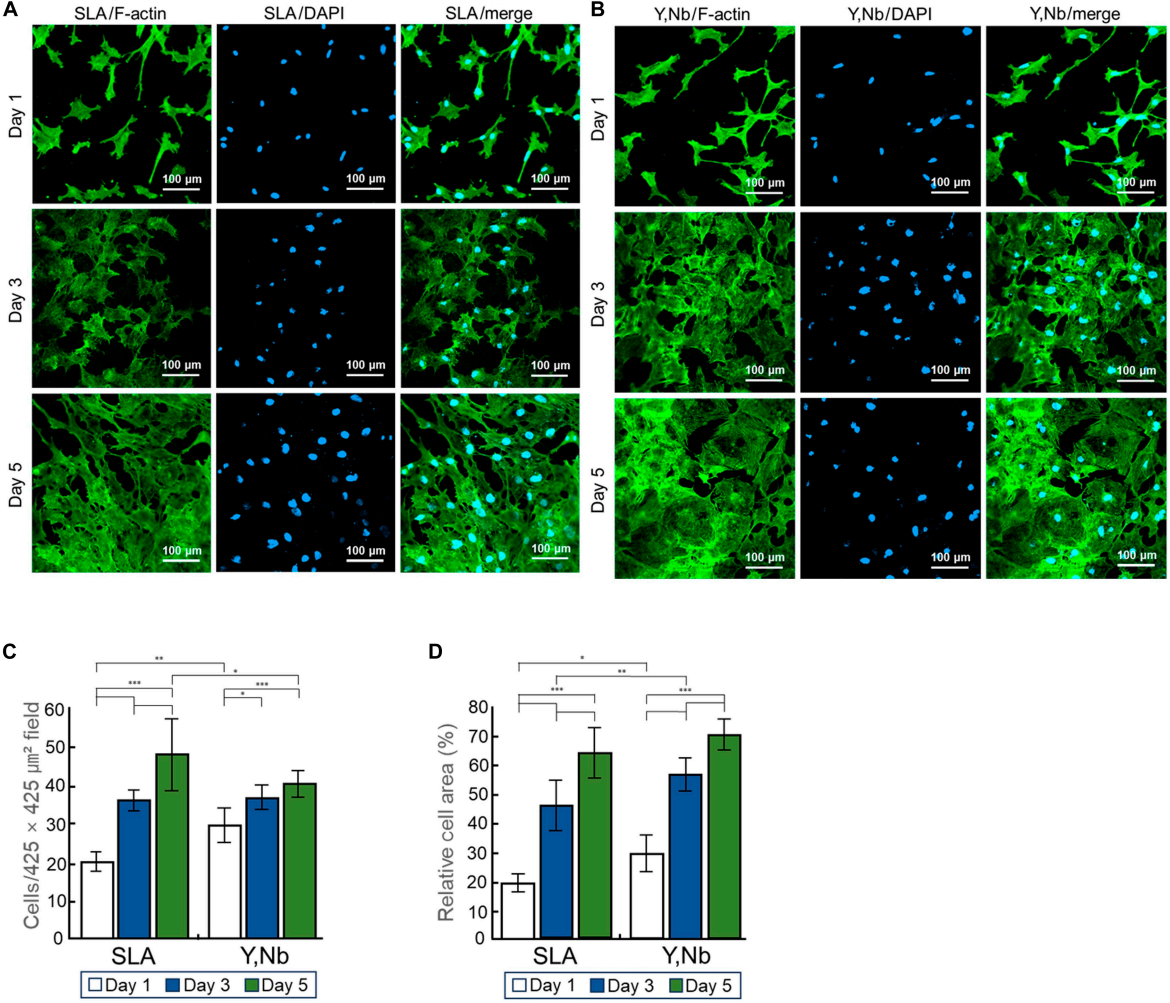
In vitro cellular responses on sandblasted, large-grit, acid-etched (SLA) titanium and yttria–niobia-stabilized tetragonal zirconia polycrystal ((Y,Nb)-TZP) surfaces. (A) Fluorescence microscopy images show the cytoskeletal organization and nuclear distribution of cells cultured on SLA titanium surfaces at days 1 (top), 3 (middle), and 5 (bottom), with F-actin stained in green (first column) and nuclei counterstained with 4′,6-diamidino-2-phenylindole (DAPI) in blue (second column) and merged images (third column). (B) Corresponding fluorescence images of cells cultured on (Y,Nb)-TZP surfaces are presented at the same time points to enable direct comparison with the SLA titanium group. (C) Quantitative analysis of cell number per unit area is presented as bar graphs. (D) Relative cell area is presented as bar graphs. For both quantitative analyses, data are presented as mean ± standard deviation (*n* = 3; *P < 0.05, **P < 0.01, and ***P < 0.001).

On the cell attachment and spreading of the MC3T3-E1 cells over 1, 3, and 5 d, CLSM images exhibited progressively increasing cell numbers and spreading areas on both the SLA titanium and (Y,Nb)-TZP surfaces (Fig. 3A and B). Specifically, on day 1, the (Y,Nb)-TZP group showed a significantly higher cell attachment count than the SLA group. However, this difference in cell count became insignificant by day 3. By day 5, the SLA titanium surface showed a higher number of attached cells (Fig. 3C). The (Y,Nb)-TZP group showed a significantly larger cell spreading area on days 1 and 3, but no significant difference in the cell spreading area was observed between the 2 groups on day 5 (Fig. 3D).

### Cell proliferation and osteoblastic differentiation

**Fig. 4.**
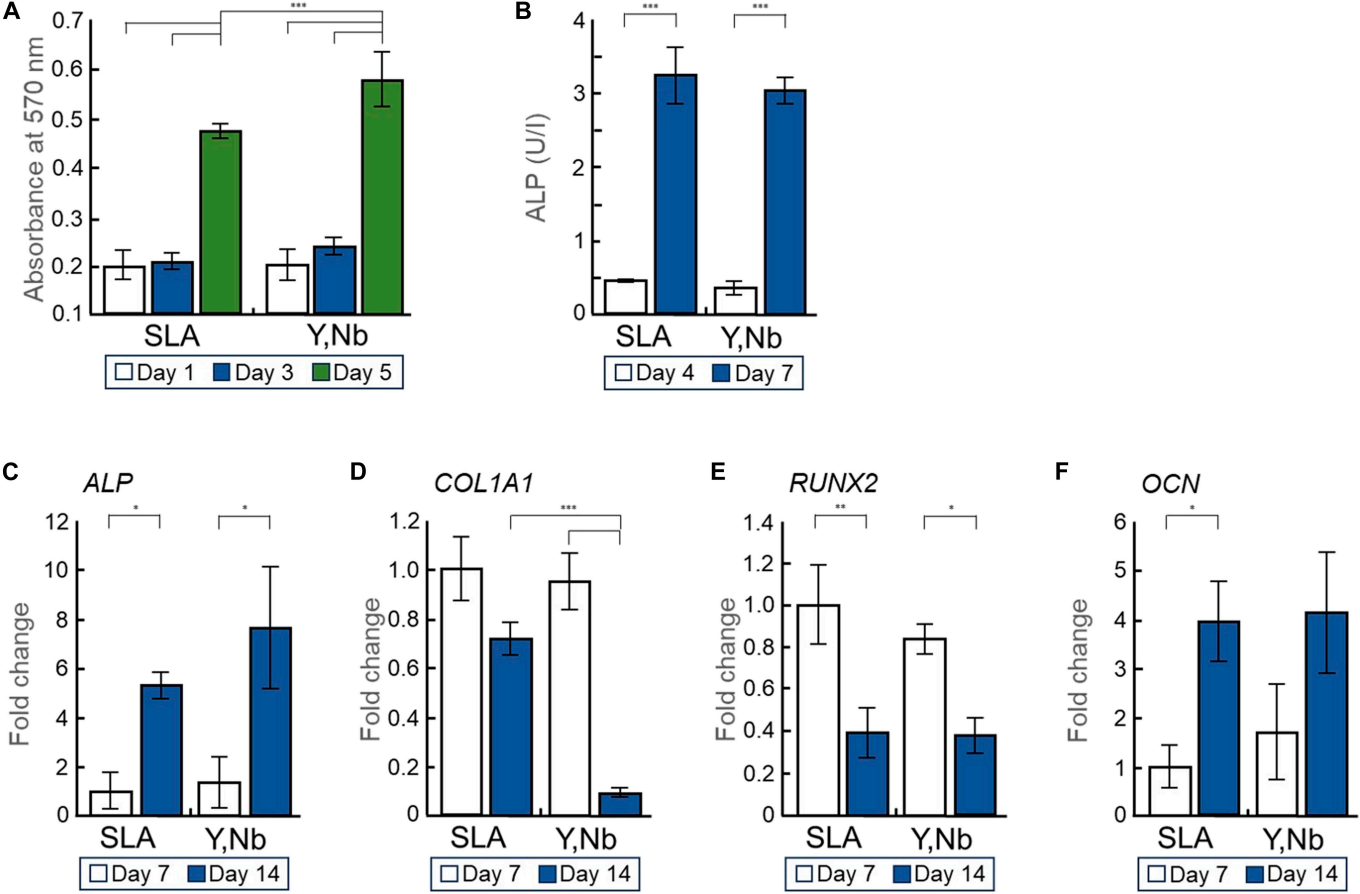
In vitro cell proliferation, osteoblastic differentiation, and osteogenic gene expression on sandblasted, large-grit, acid-etched (SLA) titanium and yttria–niobia-stabilized tetragonal zirconia polycrystal ((Y,Nb)-TZP) surfaces. (A) Cell proliferation was evaluated using the 3-(4,5-dimethylthiazol-2-yl)-2,5-diphenyltetrazolium bromide (MTT) assay at days 1, 3, and 5 of culture. (B) Osteoblastic differentiation was assessed by alkaline phosphatase (ALP) activity assay after 4 and 7 d of incubation. (C to F) Gene expression related to osteogenesis, including ALP, runt-related transcription factor 2 (RUNX2), collagen type I alpha 1 (COL1A1), and osteocalcin (OCN), was evaluated by reverse transcription quantitative real-time polymerase chain reaction (RT-qPCR) after 7 and 14 d of incubation. Gene expression levels were normalized to glyceraldehyde-3-phosphate dehydrogenase (GAPDH) and expressed as fold changes relative to the SLA titanium group. For all quantitative analyses, data are presented as mean ± standard deviation (*n* = 3; *P < 0.05, **P < 0.01, and ***P < 0.001).

Cell proliferation was assessed using the MTT assay on days 1, 3, and 5. Both the SLA titanium and (Y,Nb)-TZP groups showed a significant increase in cell viability on day 5 compared to that on days 1 and 3, indicating sustained cell proliferation over time (Fig. 4A). Notably, the (Y,Nb)-TZP group consistently exhibited significantly higher MTT values than the SLA titanium group on day 5. To assess osteoblastic differentiation, ALP activity was measured on days 4 and 7 (Fig. 4B). Both groups demonstrated a significant increase in ALP activity on day 7 compared to that on day 4, reflecting progressive osteoblastic differentiation. However, unlike cell proliferation, there was no significant difference in ALP activity between the SLA titanium and (Y,Nb)-TZP groups at any time point.

### RT-qPCR

RT-qPCR analysis was performed on days 7 and 14 to evaluate the gene expression levels of the osteogenic markers ALP, COL1A1, OCN, and RUNX2 (Fig. 4C to F). Although ALP gene expression increased on day 14 in both the SLA titanium and (Y,Nb)-TZP groups, with no statistically significant intergroup differences, COL1A1 exhibited a decreasing trend across both groups, showing a statistically significant reduction, specifically in the (Y,Nb)-TZP group. Furthermore, RUNX2 expression was significantly down-regulated on day 14 in both groups. For OCN, an increasing trend was generally observed; however, only the SLA titanium group showed a statistically significant increase on day 14, with no statistically significant intergroup differences.

### Histomorphometry and fluorescence

**Fig. 5.**
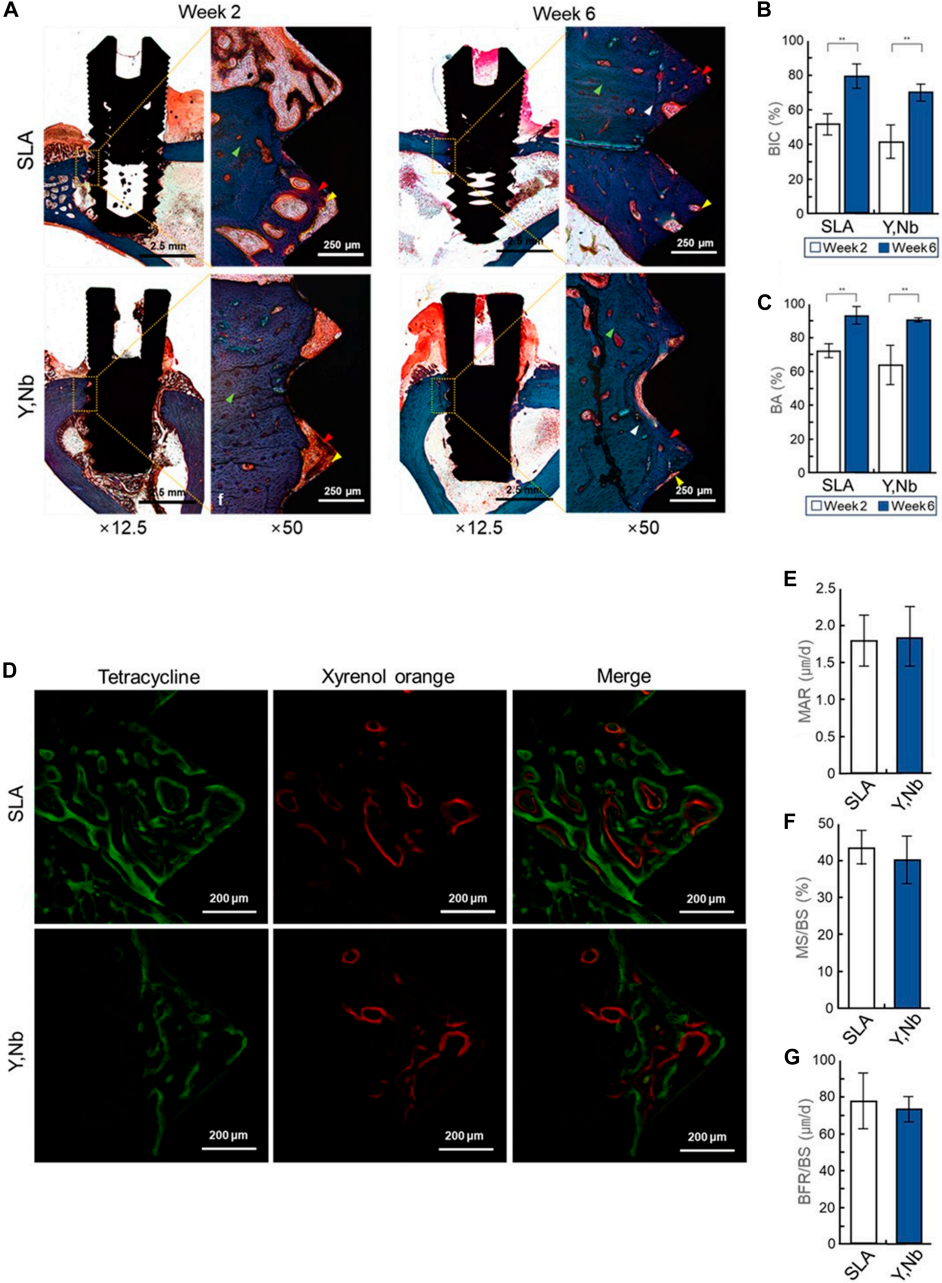
Histomorphometric and fluorescence analyses of peri-implant bone responses on sandblasted, large-grit, acid-etched (SLA) titanium and yttria–niobia-stabilized tetragonal zirconia polycrystal ((Y,Nb)-TZP) implants. (A) Representative histomorphometric images obtained at weeks 2 (first and second column) and 6 (third and fourth columns) illustrate peri-implant bone tissue responses. Newly formed bone (red arrowheads) is stained red, mature bone (green arrowheads) is stained purplish blue, reversal lines (white arrowheads) appear deep dark blue, and connective tissue (yellow arrowheads) is stained orange. (B and C) Quantitative histomorphometric parameters, including bone-to-implant contact (BIC) and bone area (BA) ratio, are presented. (D) Representative fluorescence microscopy images acquired at week 6 demonstrate dynamic bone formation around the implants. The visually weaker green signal of the (Y,Nb)-TZP group is merely an optical artifact caused by imaging adjustments using a filter to mask the fluorescence-like signal of (Y,Nb)-TZP, which did not affect the following quantitative data. (E to G) Bone remodeling indices derived from fluorescence analysis are presented. For all quantitative analyses, data are presented as mean ± standard deviation (*n* = 3; **P < 0.01).

Conventional 2D histomorphometric analysis was conducted on undemineralized sections stained with Goldner’s Masson trichrome at 2 and 6 weeks postimplantation (Fig. 5A). At 2 weeks, both the SLA titanium and (Y,Nb)-TZP implant groups demonstrated the initiation of new bone synthesis along with the presence of connective tissue between the mature bone and implant surface. By 6 weeks, the newly formed bone occupied most of the available spaces in both groups, with evident reversal lines between the new and mature bones. Quantitatively, BIC and BA significantly increased from 2 to 6 weeks in both groups, whereas no statistically significant differences in either BIC or BA were observed between the 2 groups at either time point (Fig. 5B and C).

Dynamic bone formation and remodeling were assessed using fluorescently labeled 2D sections (Fig. 5D). Tetracycline (green fluorescence), administered at 2 weeks, was predominantly observed in the space between the mature bone and implant surface, as well as on the mature bone and implant surfaces. Xylenol orange (red fluorescence) injected at 4 weeks was detected within the new bone formed around the 2-week tetracycline label, often appearing as distinct concentric rings in both groups. Quantitative assessments of MAR, MS/BS, and BFR/BS using these dynamic labels revealed no statistically significant differences between the SLA titanium and (Y,Nb)-TZP groups (Fig. 5E to G).

**Fig. 6.**
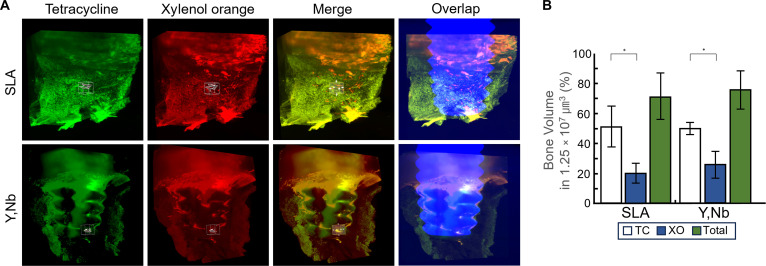
Three-dimensional (3D)-based evaluation and volumetric analysis of peri-implant bone remodeling using polyethylene glycol (PEG)-associated solvent system (PEGASOS) fluorescence imaging. (A) Representative 3D reconstructed images of peri-implant bone at 6 weeks postimplantation obtained by light-sheet fluorescence microscopy following PEGASOS-based hard tissue clearing. From left to right, images represent tetracycline-labeled bone (TC, green; 2 weeks), xylenol orange-labeled bone (XO, red; 4 weeks), merged TC and XO signals, and the merged fluorescence signals overlaid with the corresponding micro-computed tomography (micro-CT) reconstruction to illustrate the spatial relationship between dynamic bone formation and implant geometry. (B) 3D volumetric quantification of TC-labeled bone, XO-labeled bone, and total fluorescent bone volume within regions of interest (ROIs) located in the peri-implant thread grooves. Data are presented as mean ± standard deviation (*n* = 3; *P < 0.05).

For a comprehensive 3D evaluation using the PEGASOS hard-tissue-clearing method, a 3D volumetric analysis of dynamic bone remodeling was performed. Unlike titanium, (Y,Nb)-TZP exhibits laser-induced fluorescence-like signals on both laser emission filters. Qualitatively, SLA titanium implants exhibited thin and elongated fluorescent signals that were discontinuously distributed along the implant threads. In contrast, the (Y,Nb)-TZP implants demonstrated fluorescent signals with a more concentrated appearance that was spatially localized and appeared as clustered aggregates in specific regions of the threads (Fig. 6A). Furthermore, to allow for a direct comparison with traditional 2D histology, sequential 2D slices were extracted from these 3D volumes, enabling a comprehensive layer-by-layer observation (Movies S1 and S2). The volumes of the 2-week tetracycline and 4-week xylenol orange labels in the subcortical regions around the implants revealed that the proportion of the 2-week fluorescent labels was higher than that of the 4-week labels in both groups. Consistent with the 2D dynamic histomorphometry results, no statistically significant differences in the 3D dynamic remodeling volumes were observed between the SLA titanium and (Y,Nb)-TZP groups (Fig. 6B).

## Discussion

In this study, sandblasted (Y,Nb)-TZP demonstrated comparable in vitro osteogenic potential and in vivo osseointegration to conventional SLA titanium, despite their distinct differences in material composition and surface characteristics. Sandblasting on a zirconia surface is known to generate appropriate roughness for osseointegration while simultaneously inducing topographical and crystalline changes that can improve mechanical properties such as flexural strength and shear bond strength [26,27]. Consistent with our findings, previous studies reported that the incorporation of yttrium and niobium can enhance the biological performance of zirconia [16,17,22,28]. The overall results of this study indicate that (Y,Nb)-TZP can promote peri-implant bone formation and remodeling, suggesting its potential clinical applicability.

In this study, 3D visualization of peri-implant bone remodeling was performed on the cortical bone area using the PEGASOS hard-tissue-clearing technique. The 3D-based analysis visualizes unique subcortical bone dynamics that are difficult to evaluate using traditional methods, whereas conventional 2D histomorphometry sufficiently demonstrates peri-implant bone responses around the cortical bone regions [29,30]. Interestingly, fluorescence-like signals were observed on the (Y,Nb)-TZP implant surfaces under both laser channels during laser-based 3D imaging, whereas no such signals were detected on SLA titanium. Similar to our results, previous studies have reported that zirconia-based ceramics can exhibit laser-induced optical emissions under specific excitation conditions, suggesting the possibilities of intrinsic material-dependent signals [31,32]. Specifically, because 3D volumetric imaging captures a much larger mass of the bulk material compared to thin 2D sections, this accumulated fluorescence-like signal of (Y,Nb)-TZP becomes significantly stronger. Consequently, even with the application of optical filters, the residual signal remains intense enough to visually outline the macroscopic threadlike structure of the (Y,Nb)-TZP implant, as observed in Fig. 6. These findings highlight the need for careful interpretation of fluorescence-based images using specific filters or adjustment of thresholds when evaluating ceramic implants. In 3D-based volumetric analysis, the volume ratio of tetracycline-labeled bone (2 weeks) was significantly greater than that of xylenol orange-labeled bone (4 weeks) in both groups. Considering the rapid bone turnover rate in rabbits compared to that in humans, the 2-week time point corresponds to an early and burst-like phase of new bone formation [33,34]. Bone maturation around the previously formed bone occurred at the 4-week time point, resulting in a relatively small volume of new bone formation [35]. Although the overall volume ratio of dynamic bone remodeling was similar between SLA titanium and (Y,Nb)-TZP implants, 3D visualization suggested different spatial distributions of fluorescently labeled bones along implant threads [36]. The relatively localized but intense bone formation observed around (Y,Nb)-TZP implants may reflect material-specific interactions within the bone marrow environment, which is biologically distinct from the cortical bone area, although the exact biological mechanisms require further investigation [35,37,38].

Although the surface roughness of SLA titanium was significantly higher than that of (Y,Nb)-TZP, the zirconia implants exhibited comparable in vitro and in vivo results [17,22]. In a previous study, modified rough surfaces showed a more favorable bone response than unmodified smooth surfaces for both zirconia and titanium [39]. Therefore, we consider adequate surface roughening, such as the sandblasting treatment, an essential requisite for zirconia implants to achieve rapid and predictable clinical osseointegration comparable to the current titanium gold standard. The surface roughness of (Y,Nb)-TZP (approximately 1 to 1.6 μm) falls within the range commonly defined as moderately rough, which has been reported to be favorable for osteoblastic attachment and function rather than excessively rough surfaces [40,41]. In this context, there was significantly higher cell attachment to (Y,Nb)-TZP on day 1, together with a larger cell spreading area on days 1 and 3 [42]. These results suggest that the zirconia surface provides a more favorable environment for initial cell attachment, which may be critical for early events that influence subsequent cellular behavior [43]. Although the number of cells on (Y,Nb)-TZP became lower than that on SLA titanium at day 5, the absence of a significant difference in cell spreading area indicates that individual cells on zirconia maintained comparable cytoskeletal extension [44]. This discrepancy between cell number and area suggests that a reduced cell density does not necessarily reflect diminished cellular activity [45]. Consistently, the higher MTT values observed for (Y,Nb)-TZP on day 5 imply enhanced cellular metabolic activity, suggesting that zirconia may sustain active cellular function despite differences in cell number [46].

At the cellular level, the osteogenic responses to SLA titanium and (Y,Nb)-TZP were comparable. Both groups exhibited a significant increase in ALP activity over time without intergroup differences, indicating stable osteoblastic differentiation [47]. Consistent with this result, ALP expression, as assessed by RT-qPCR, also increased with culture time in both groups, with no statistically significant intergroup differences, demonstrating an agreement between functional enzymatic activity and transcriptional regulation. Furthermore, other RT-qPCR results showed similar patterns between the groups and revealed no significant intergroup differences in the overall expression of osteogenic genes, except for COL1A1 on day 14. RUNX2 and COL1A1, early osteogenic markers, showed a gradual decrease, whereas the late-stage marker OCN exhibited an increasing tendency, reflecting normal progression from early differentiation to matrix maturation [48]. Although COL1A1 expression in (Y,Nb)-TZP decreased more markedly than in SLA titanium on day 14, this tendency is consistent with previous studies that reported temporal down-regulation of early matrix genes during osteoblast maturation on zirconia surfaces [17]. These results indicate that both materials promote the stable and coordinated expression of osteogenesis-related genes. These in vitro findings were further supported by in vivo analyses, in which histomorphometric parameters, including BIC and BA, showed no significant differences between groups [22,39,49]. In addition, fluorescence-based dynamic bone remodeling indices, including MAR, MS/BS, BFR/BS, and bone volume ratio, demonstrated comparable values in both 2D and 3D analyses [50]. These results reinforce the idea that (Y,Nb)-TZP and SLA titanium induce similar peri-implant bone formation and remodeling responses under the present experimental conditions.

This study has some limitations. A formal a priori power analysis was not conducted, and the sample size was determined based on previous comparable studies to minimize animal use. Thus, studies with larger sample sizes are required to statistically strengthen the current findings. Because decalcification was omitted to preserve the fluorochrome labeling signals and the structure of the subcortical and bone marrow regions, detailed visualization of the cortical bone structures was not feasible in the 3D-cleared specimens. Moreover, the macrostructural differences arising from different fabrication procedures and hard-tissue-clearing protocols for larger animal models, such as rabbits, should be standardized. Additionally, although our bone remodeling indices (MAR, MS/BS, and BFR/BS) provided valuable insights into the rate of bone formation and early mineralization activity, a comprehensive mineralization analysis to thoroughly assess the degree of bone maturation at different time points was not conducted. Further studies incorporating detailed temporal assessments of bone maturation, long-term functional loading, optimized clearing strategies that sustain both mineral preservation and fluorescence retention, and diverse animal models are needed to evaluate the biological mechanisms of (Y,Nb)-TZP in bone interactions and strengthen its clinical relevance.

This study demonstrated that the novel (Y,Nb)-TZP implant exhibited biocompatibility and osseointegration comparable to those of clinically established SLA titanium. Although conventional in vitro and histomorphometric analyses revealed no significant differences between the 2 materials, 3D-based hard-tissue-clearing analysis provided additional information that could not be obtained using traditional methods. These findings support that (Y,Nb)-TZP has profound capabilities for peri-implant bone remodeling performance and highlight the value of 3D-based analysis in evaluating implant–bone interactions beyond conventional 2D assessments.

## Data Availability

All relevant data are available from the corresponding authors upon reasonable request.
